# First‐line treatment with irreversible tyrosine kinase inhibitors associated with longer OS in *EGFR* mutation‐positive non‐small cell lung cancer

**DOI:** 10.1111/1759-7714.13462

**Published:** 2020-12-18

**Authors:** Po‐Lan Su, Chian‐Wei Chen, Yi‐Lin Wu, Chien‐Chung Lin, Wu‐Chou Su

**Affiliations:** ^1^ Department of Internal Medicine, National Cheng Kung University Hospital, College of Medicine National Cheng Kung University Tainan Taiwan; ^2^ Department of Nursing, National Cheng Kung University Hospital, College of Medicine National Cheng Kung University Tainan Taiwan; ^3^ Department of Internal Medicine and Institute of Clinical Medicine, College of Medicine National Cheng Kung University Hospital Tainan Taiwan

**Keywords:** EGFR‐TKI, non‐small cell lung cancer, progression‐free survival

## Abstract

**Background:**

Few studies have compared the efficacy of the irreversible epidermal growth factor receptor tyrosine kinase inhibitor (EGFR‐TKI), afatinib, with that of reversible EGFR‐TKIs. Therefore, this study assessed the effectiveness of afatinib, erlotinib, and gefitinib in terms of OS (overall survival) and progression‐free survival (PFS) in *EGFR* mutation‐positive advanced non‐small cell lung cancer (NSCLC) patients.

**Methods:**

Patients with *EGFR* mutation‐positive advanced NSCLC who sought treatment from December 2013 to June 2018, at a tertiary referral center were retrospectively analyzed. These patients were treated with afatinib or a reversible EGFR‐TKI (erlotinib or gefitinib) until disease progression, intolerable adverse events, or death. The Kaplan‐Meier and log‐rank tests were then used to compare the OS and PFS of the patients. We further analyzed the survival differences among the subgroup of patients without brain metastases.

**Results:**

Of the 363 patients enrolled, 134 and 229 received first‐line afatinib and first‐line reversible EGFR‐TKI, respectively. Those given afatinib had better OS (39.3 vs. 26.0 months; HR 0.65, *P* = 0.033) and PFS (14.1 vs.11.2 months; HR 0.58, *P* < 0.001). Of the 246 patients without brain metastases, 93 and 153 received first‐line afatinib and a first‐line reversible EGFR‐TKI, respectively. Those given afatinib had a better OS (52.6 vs. 24.9 months; HR 0.62, *P =* 0.0030) and PFS (17.7 vs. 11.1 months; HR 0.51, *P* < 0.001). The survival benefit was more significant in the subgroup of patients with L858R substitutions.

**Conclusions:**

The results indicated that afatnib resulted in significantly better OS and PFS than gefitnib and erlotinib for *EGFR* mutation‐positive advanced NSCLC patients without brain metastases.

**Key points:**

**Significant findings of the study**

Afatnib resulted in significantly better overall survival and progression‐free survival than gefitnib and erlotinib for *EGFR* mutation‐positive advanced non‐small cell lung cancer patients without brain metastases.

**What this study adds**

This study helps fill the gap in our limited understanding of the differences in the efficacy of the irreversible epidermal growth factor receptor tyrosine kinase inhibitor (EGFR‐TKI), afatinib, with that of reversible EGFR‐TKIs.

## Introduction

The epidermal growth factor receptor (*EGFR*) mutations are the most common oncogenic driver mutations in pulmonary non‐small cell lung cancer (NSCLC), having been found to occur in 11%–16% of patients in Western countries and around 50% of Asian patients.^1^ Relatedly, for patients with advanced *EGFR*‐mutant NSCLC, EGFR tyrosine kinase inhibitors (TKIs) have yielded improved objective response rates and progression‐free survival (PFS) compared with chemotherapy.^2^ However, even as EGFR‐TKIs have become a mainstay treatment, studies have revealed no difference in overall survival (OS) between chemotherapy and two reversible EGFR‐TKIs, gefitinib and erlotinib.^2^, ^3^, ^4^ On the other hand, an irreversible EGFR‐TKI, afatinib,^5^ was found to not only yield significant PFS enhancements in comparison to chemotherapy but also significantly better OS than chemotherapy according to a pooled analysis of data from the LUX‐Lung 3 and LUX‐Lung 6 phase 3 clinical trials.^6^, ^7^ More recently, the FLAURA study revealed that another irreversible EGFR‐TKI, osimertinib, also yields better PFS and OS than the reversible EGFR‐TKIs gefitinib and erlotinib, especially in patients with central nervous system metastasis.^8^, ^9^

Meanwhile, both the LUX‐Lung 7 study and ARCHER 1050 study demonstrated that two second‐generation irreversible EGFR‐TKIs, afatinib and dacomitinib, could provide significantly improved PFS compared with gefitinib.^10^, ^11^ An OS benefit over gefitnib was also reported in the ARCHER 1050 clinical trial of dacomitinib, which excluded patients with brain metastases (BM).^12^ Moreover, in a subgroup analysis of the LUX‐Lung 7 study, afatinib was found to provide better OS than gefitinib in the absence of BM.^13^ Taken together, the above findings indicate not only that the reversible and irreversible EGFR‐TKIs have different levels of efficacy in general, but that the irreversible EGFR‐TKIs may be particularly beneficial, comparatively, in terms of survival in patients without BM. In the current real‐world study, therefore, we compared the OS and PFS of patients with *EGFR*‐mutant advanced NSCLC treated with the irreversible EGFR‐TKI afatinib or with one of two reversible EGFR‐TKIs, erlotinib or gefitinib, while also conducting a subgroup analysis among patients without BM.

## Methods

In our previous retrospective real‐world study, we compared the PFS and OS among patients treated with different EGFR‐TKIs between 1 December 2013, and 30 November 2017.^14^ However, the patient population in that study was insufficient to perform any subgroup analyses. Thus, we also included patients treated from 1 December 2017, to 30 June 2018, in the present study for a pooled analysis. This study was reviewed and approved by the Review Board and Ethics Committee of National Cheng Kung University Hospital (NCKUH B‐ER‐109–043). All data were anonymized, and, given the retrospective nature of the study, the need for written informed consent was waived. All *EGFR*‐mutation positive patients with recurrent or newly diagnosed advanced NSCLC who were treated at National Cheng Kung University Hospital between 1 December 2013, and 30 June 2018, were enrolled in the study, except for those with *EGFR* mutations other than Del 19 and L858R mutations, who were excluded. All the patients underwent a chest computed tomography (CT) scan, bone scan, and brain imaging (CT or magnetic resonance imaging) scan for staging based on the tumor, node, metastasis (TNM) classification proposed by the American Joint Committee on Cancer, eighth edition. Stage I–IIIA patients were excluded, such that only advanced‐stage patients were ultimately included in the analysis.

We recorded the baseline characteristics of these patients, including age, sex, mutation subtypes, performance status, BM status, and TNM staging. All of the patients took either a reversible EGFR‐TKI (gefitinib or erlotinib) or an irreversible EGFR‐TKI (afatinib) at the discretion of the treating providers. The treatment modalities including these EGFR‐TKIs and subsequent therapies, such as osimertinib, were recorded. Disease progression was determined based on radiographic evidence according to Response Evaluation Criteria in Solid Tumors (RECIST) version 1.1.

### *EGFR* mutation analysis

Tumor tissues from primary lung tumors or metastatic lesions were obtained for EGFR mutation analysis. Tissue samples that consisted of >80% tumor content, as determined via microscopy, were selected for the study. DNA was extracted using the QIAcube automated extractor (Qiagen, Hilden, Germany) with the QIAamp DNA FFPE tissue kit (Qiagen) and eluted in ATE (QIAmp Tissue Elution) buffer (Qiagen), according to the manufacturer's instructions. The presence of *EGFR* mutations was determined using the EGFR PCR Kit (EGFR RUO Kit) and therascreen EGFR RGQ PCR Kit (EGFR IVD Kit, Qiagen, Manchester, UK). These kits combine the Scorpions and amplification‐refractory mutation system (ARMS) technologies to detect mutations using real‐time quantitative PCR.^15^

### Statistical analysis

Demographic and clinical variables were compared using the Chi‐square test or Fisher's exact test for categorical variables and Student's *t*‐test for continuous variables. The PFS and OS of the total patients and the patients without initial BM were estimated by the Kaplan‐Meier method and compared by the log‐rank test. The definition of BM included any parenchymal BM and radiographically diagnosed leptomeningeal disease. We also performed Cox proportional hazards regression for the determinants of PFS and OS among the patients without initial BM. The selection of possible determinants was based on prior studies investigating the risk factors for the prognostic factors of survival.^16^, ^17^ Age, sex, post‐operative recurrence, performance status, tumor size, nodal stage, and *EGFR* mutation subtypes were chosen as possible prognostic factors. Statistical Analysis System software version 9.4 (SAS Institute, Cary, North Carolina, USA) was used to perform the analyses. All the reported *P*‐values are two‐sided.

## Results

### Patient characteristics

**Figure 1:**
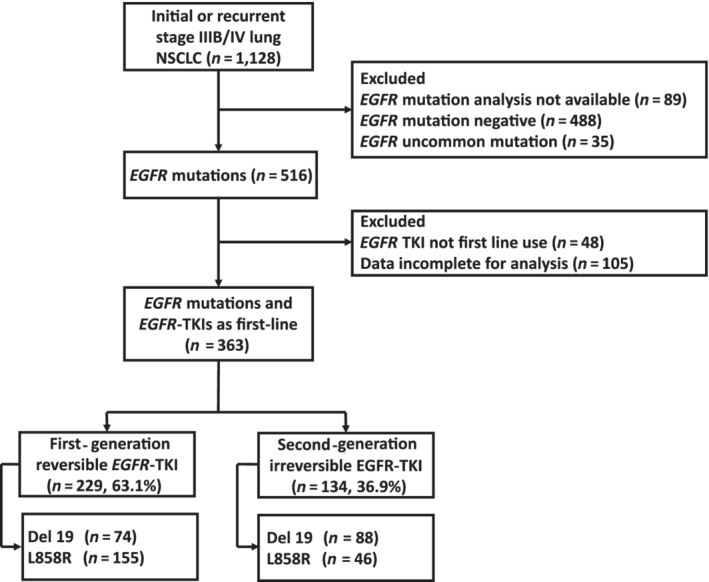
Study flow chart.

A total of 363 patients were enrolled. Among those patients, 229 patients received a reversible EGFR‐TKI, gefitinib or erlotinib, and 134 patients received the irreversible EGFR‐TKI afatinib as the first‐line therapy. The patients who received the irreversible EGFR‐TKI had higher proportions of Del 19 mutations and performance statuses of 0–1. The detailed patient characteristics of all 363 patients are shown in Table 1. In the subgroup of patients without BM, 153 patients received a reversible EGFR‐TKI and 93 patients received the irreversible EGFR‐TKI as the first‐line therapy. In this subgroup of patients without BM, those who received the irreversible EGFR‐TKI were younger on average and had higher proportions of Del 19 mutations and performance statuses of 0–1. The detailed patient characteristics of this subgroup are shown in Table S1. Figure 1 details the inclusion of subjects for analysis.

**Table 1:** Demographic and clinical characteristics of the overall patient cohort, subdivided by those treated with the first‐generation EGFR‐TKIs and those treated with the second‐generation EGFR‐TKI

	First‐generation (*N* = 229)	Second‐generation (*N* = 134)	*P*‐value
Age	69.3 (60.6–78.4)	65.2 (56.2–75.0)	0.230
Sex, *n* (%)			0.926
Male	90 (39.3%)	52 (38.8%)	
Female	139 (60.7%)	82 (61.2%)	
Tumor size, *n* (%)			0.379
≥ 3cm	157 (68.6%)	94 (70.1%)	
< 3cm	51 (22.3%)	33 (24.6%)	
NA	21 (9.1%)	7 (5.3%)	
Nodal involvement, *n* (%)			0.903
N0	32 (14.0%)	18 (19.4%)	
N1/N2/N3	197 (86.0%)	75 (80.6%)	
Stage, *n* (%)			0.705
Recurrence	26 (11.4%)	21 (15.7%)	
Newly‐diagnosed	203 (88.6%)	113 (84.3%)	
ECOG PS, *n* (%)			0.005
0–1	187 (81.7%)	121 (90.3%)	
≥ 2	42 (18.3%)	13 (9.7%)	
*EGFR* mutation, *n* (%)			<0.001
Del 19	74 (32.3%)	88 (65.7%)	
L858R	155 (67.7%)	46 (34.3%)	

ECOG, Eastern Cooperative Oncology Group; EGFR, epidermal growth factor receptor; PS, performance status; NA, not applicable.

### PFS and OS of total population

**Figure 2:**
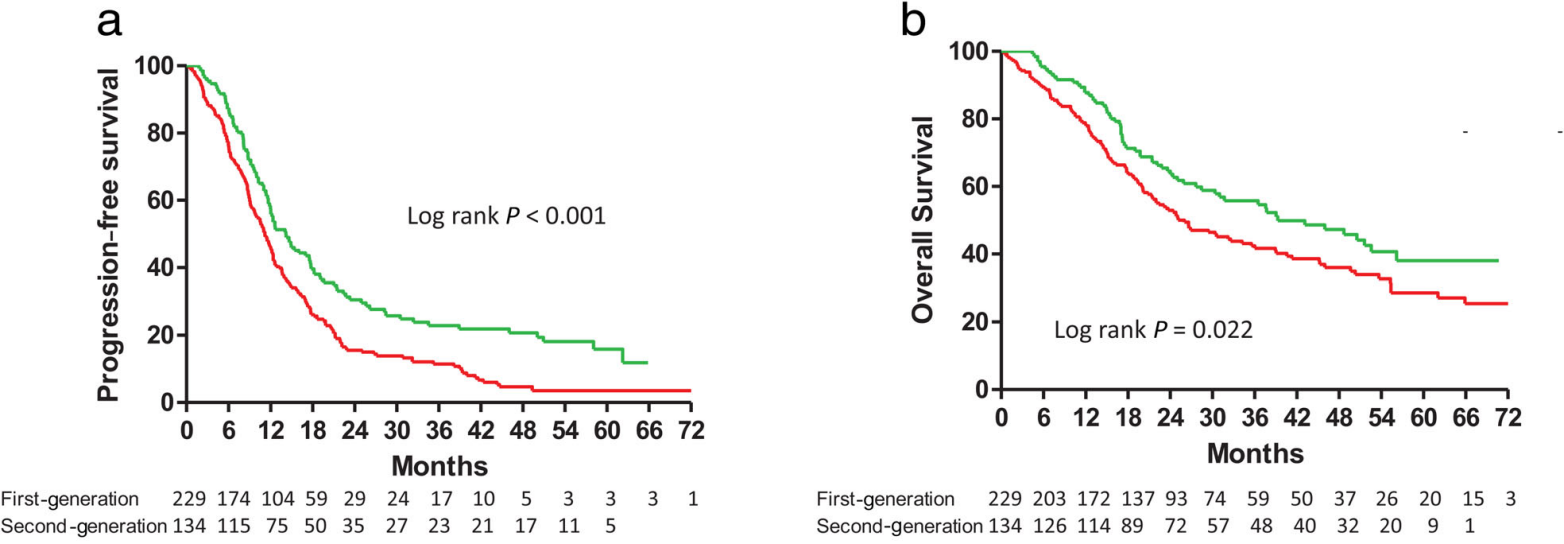
(**a**) Progression‐free survival (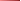) First‐generation 11.2 (6.1–18.6) (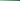) Second‐ generation 14.1 (8.6–30.5); and (**b**) Overall survival in patients with advanced stage non‐small cell lung cancer and epidermal growth factor receptor gene mutations treated with first‐ or second‐generation EGFR‐TKIs (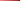) First‐generation 26.0 (12.9–NR) (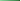) Second‐generation 39.3 (17.2–NR).

A comparison of the PFS and OS of all the patients stratified by the different types of EGFR‐TKIs is shown in Figure 2. The patients receiving the reversible and irreversible EGFR‐TKIs had median PFS durations of 11.2 and 14.1 months, respectively. Furthermore, the patients receiving the reversible and irreversible EGFR‐TKIs had median OS durations of 26.0 and 39.3 months, respectively. Both PFS and OS were significantly longer in the irreversible EGFR‐TKI group than in the reversible EGFR‐TKI group (Fig 2a,b).

**Figure 3:**
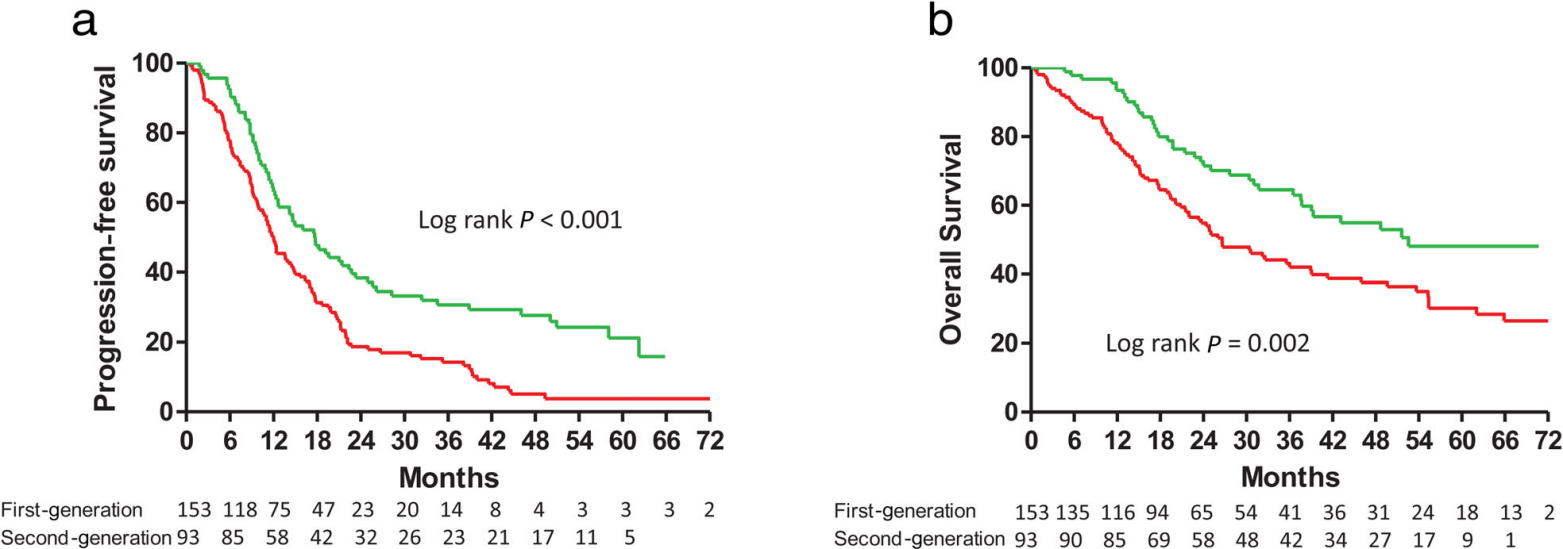
(**a**) Progression‐free survival (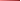) First‐generation 12.0 (6.3–21.2) (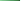) Second‐generation 17.7 (9.7–50.9); and (**b**) Overall survival in patients with advanced stage non‐small cell lung cancer and epidermal growth factor receptor gene mutations, but without brain metastases, treated with first‐ or second‐generation EGFR‐TKIs (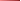) First‐generation 56.2 (22.8‐NR) (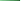) Second‐generation 26.6 (13.1–NR).

In the subgroup of patients without BM, the patients receiving the reversible and irreversible EGFR‐TKIs had median PFS durations of 12.0 and 17.7 months, respectively. That is, the PFS was also longer for the subgroup patients receiving the irreversible EGFR‐TKI than for the subgroup patients receiving the reversible EGFR‐TKIs (Fig 3a). Using Cox proportional hazards regression to adjust for possible confounders, we found that the hazard ratio (HR) of PFS for the irreversible versus reversible EGFR‐TKIs was 0.58 (95% CI: 0.43–0.80, *P* < 0.001, Table 2). Other independent predictors for better PFS were female gender, good performance status, and negative nodal involvement (Table 2).

**Table 2:** Cox proportional hazards regression results indicating progression‐free survival and overall survival of patients without brain metastases

		Progression‐free survival		Overall survival	
		HR (95% CI)	*P‐* value	HR (95% CI)	*P‐* value
Age	≥60 vs. <60	0.89 (0.64–1.24)	0.501	1.33 (0.87–2.04)	0.194
Sex	Male vs. female	1.47 (1.11–1.95)	0.007	1.59 (1.11–2.27)	0.011
ECOG PS	≥ 2 vs. < 2	2.58 (1.59–4.18)	<0.001	4.30 (2.45–7.53)	<0.001
Tumor size	>3 cm vs. < 3 cm	1.37 (0.96–1.94)	0.080	1.85 (1.15–2.97)	0.012
Nodal involvement	Positive versus negative	1.98 (1.31–3.00)	0.001	1.86 (1.10–3.16)	0.022
*EGFR* mutation	Del 19 vs. other mutation	1.11 (0.83–1.48)	0.484	1.01 (0.70–1.46)	0.950
Recurrence	Newly‐diagnosed vs. recurrence	0.97 (0.64–1.48)	0.892	1.03 (0.59–1.79)	0.918
Treatment	Second‐generation vs. first‐generation	0.58 (0.43–0.80)	<0.001	0.65 (0.44–0.97)	0.033

ECOG, Eastern Cooperative Oncology Group; EGFR, epidermal growth factor receptor; PS, performance status.

Moreover, the patients receiving the reversible and irreversible EGFR‐TKIs had median OS durations of 26.6 and 52.6 months, respectively. That is, the OS was also longer for the subgroup patients receiving the irreversible EGFR‐TKI than for the subgroup patients receiving the reversible EGFR‐TKIs (Fig 3b). Using Cox proportional hazards regression to adjust for possible confounders, we found that the HR of OS for the irreversible versus the reversible EGFR‐TKIs was 0.62 (95% CI: 0.41–0.96, *P* = 0.030). Other independent predictors for better OS included female gender, good performance status, negative nodal involvement, and smaller tumor size (Table 2).

### PFS and OS of patients harboring different *EGFR* mutations

**Figure 4:**
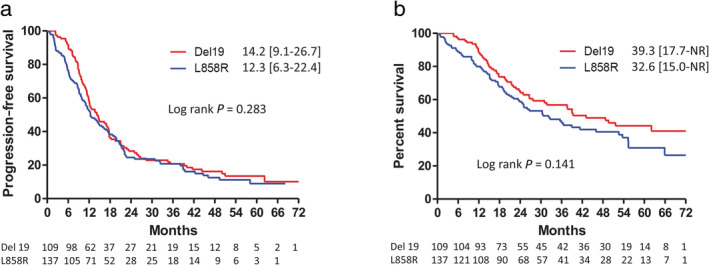
(**a**) Progression‐free survival (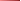) Del 19 14.2 (9.1–26.7) (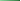) L858R 12.3 (6.3–22.4); and (**b**) Overall survival in patients with advanced stage non‐small cell lung cancer and epidermal growth factor receptor gene mutations (either Del 19 or L858R), but without brain metastases, treated with EGFR‐TKIs (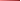) Del 19 39.3 (17.7–NR) (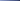) L858R 32.6 (15.0–NR).

For advanced NSCLC patients, whether exon 19 deletions are associated with longer PFS compared to L858R mutations after treatment with first‐line EGFR‐TKIs remains controversial.^18^, ^19^ In the current study, the patients harboring Del 19 and L858R mutations had median PFS durations of 14.2 months and 12.3 months, respectively. No significant difference between these two groups was noted (Fig 4a). The patients harboring Del 19 mutations had a median OS of 39.3 months, which was better than that for the patients harboring L858R mutations (32.6 months), but not to a statistically significant degree (Fig 4b). Using Cox proportional hazards regression to adjust for possible confounders, it was found that the mutation type was not a significant determinant of PFS or OS (Table 2).

In the patients harboring L858R substitutions, those receiving first‐generation and second‐generation EGFR‐TKIs had median PFS durations of 11.5 and 21.0 months, respectively. The PFS was significantly longer in the second‐generation EGFR‐TKI group than in the first‐generation EGFR‐TKI group (Fig 4a). Moreover, the OS for the patients receiving the second‐generation EGFR‐TKI was also significantly longer than that for the first‐generation EGFR‐TKI group (52.6 vs. 26.6 months, Fig 4b). Using Cox proportional hazards regression to adjust for possible confounders, we found that the HRs of PFS and OS for the second‐generation versus first‐generation EGFR‐TKIs were 0.53 (95% CI: 0.32–0.96, *P* = 0.011) and 0.49 (95% CI: 0.26–0.93, *P* = 0.028). Other independent predictors for better PFS and OS were female gender, good performance status, and smaller tumor size (Table S2).

In contrast, in those patients harboring exon 19 deletions, there was no significant difference in PFS or OS among the patients receiving the different types of EGFR‐TKIs (log rank *P* = 0.073 and 0.326, respectively, Fig 4c,d). Using Cox proportional hazards regression to adjust for possible confounders, we found that the use of the second‐generation EGFR‐TKI was not a predictor for better PFS or OS (Table S3).

## Discussion

To the best of our knowledge, this is the first real‐world study to report an overall survival benefit of an irreversible EGFR‐TKI compared to reversible EGFR‐TKIs in patients with advanced stage *EGFR*‐mutant NSCLC without BM.

Emerging data from the phase 3 FLAURA trial, which enrolled untreated patients with *EGFR*‐mutant NSCLC who then received osimertinib versus a reversible EGFR‐TKI (erlotinib or gefitinib), showed a better median PFS in the patients treated with osimertinib (18.9 months vs. 10.2 months; HR 0.46).^9^ Osimertinib has therefore come to be used as a standard first‐line therapy by many physicians, and has received approval from the US Food and Drug Administration (FDA) for that purpose. Recently, the FLAURA study also demonstrated an OS benefit in first‐line use of osimertinib over reversible EGFR‐TKIs (38.6 months vs. 31.8 months; HR 0.80).^8^ In the current study, the irreversible EGFR‐TKI afatinib provided longer PFS than the reversible EGFR‐TKIs gefitinib and erlotinib. The PFS with afatinib in this study was 14.1 months, which was slightly inferior to that of osimertinib in the FLAURA study (18.9 months). However, afatinib in the current study provided a median OS comparable to that with osimertinib in the FLAURA study (39.1 vs. 38.6 months). Moreover, the hazard ratio compared to the reversible EGFR‐TKIs was also similar in PFS (0.58 vs. 0.46) and OS (0.65 vs. 0.80). Taken together, these data may indicate that first‐line afatinib may have similar treatment efficacy to first‐line osimertinib. A comparison of the treatment efficacies in the current study and the LUX‐Lung 7, ARCHER 1050, and FLAURA studies is summarized in Table 3. The results of this study were different from those of the LUX‐Lung 7 study, which failed to show an OS benefit of afatinib in comparison with gefitinib.^13^ There are a number of possible reasons for this difference. First, the LUX‐Lung 7 study compared afatinib to gefitinib only. In the current study, however, we compared afatinib to pooled data for gefitinib and erlotinib.^10^, ^13^ Second, in this study, the patients who received afatinib had better performance statuses than the patients who received the reversible EGFR‐TKIs (Chi‐square test, *P* = 0.005). However, we performed a Cox proportional hazard regression analysis to eliminate possible confounders. In addition, objective responses were observed in 70% of the patients in the afatinib arm versus 56% in the gefitinib arm (*P* = 0.0083) in the LUX‐Lung 7 study, and post‐hoc analysis indicated that dose reductions effectively lightened treatment‐related adverse events without compromising the PFS benefits of afatinib.^20^ Patients receiving afatinib may benefit from tumor control and then be able to tolerate subsequent treatment. Therefore, the choice of the irreversible EGFR‐TKI afatinib may provide a survival benefit similar to that of osimertinib. Additionally, at its current cost, osimertinib is not a cost‐effective first‐line therapy for *EGFR*‐mutated NSCLC according to the World Health Organization cost‐effectiveness threshold criteria,^21^ so afatinib may be a good alternative for first‐line treatment.

**Table 3:** Comparison of current study results with those of randomized trials for patients without brain metastases

Characteristic	Current study		LUX‐lung^7^, ^10^, ^13^		ARCHER 1050^11^, ^12^		FLAURA^8^, ^9^	
EGFR‐TKI	Afatinib	Erlotinib or Gefitinib	Afatinib	Gefitinib	Dacomitinib	Gefitinib	Osimertinib	Erlotinib or Gefitinib
Total population (including patients with BM)	*n* = 134	*n* = 229	*n* = 160	*n* = 159			*n* = 279	*n* = 277
Median PFS, months HR (vs. control)	14.1 (8.6–30.5)	11.2 (6.1–18.6)	11.0 (10.6–12.9)	10.9 (9.1–11.5)			18.9 (15.2–21.4)	10.2 (9.6–11.1)
	0.65 (0.51–0.81)		0.73 (0.57–0.95)				0.46 (0.37–0.57)	
Median OS, months HR (vs. control)	39.3 (17.2–NR)	26.0 (12.9–NR)	27.9 (25.1–32.2)	24.5 (20.6–29.3)			38.6 (34.5–41.8)	31.8 (26.6–36.0)
	0.72 (0.54–0.95)		0.86 (0.66–1.12)				0.80 (0.64–1.00)	
Population without BM	*n* = 93	*n* = 153	*n* = 134	*n* = 135	*n* = 227	*n* = 225	*n* = 226	*n* = 214
Median PFS, months HR (vs. control)	17.7 (9.7–50.9)	12.0 (6.3–21.2)	12.7 (10.9–13.3)	10.9 (9.1–12.7)	14.7 (11.1–16.6)	9.2 (9.1–11.0)	19.1 (15.2–23.5)	10.9 (9.6–12.3)
	0.58 (0.43–0.80)		0.74 (0.56–0.98)		0.59 (0.47–0.74)		0.46 (0.36–0.59)	
Median OS, months HR (vs. control)	52.6 (22.8–NR)	26.6 (13.1–NR)	NA	NA	34.1 (29.5–37.7)	26.8 (23.7–32.1)	NA	NA
	0.65 (0.44–0.97)		0.81 (0.61–1.07)		0.76 (0.58–0.99)		0.79 (0.61–1.01)	

BM, brain metastases; EGFR, epidermal growth factor receptor; HR, hazard ratio; NA, not available; OS, overall survival; PFS, progression‐free survival.

In the FLAURA study, osimertinib provided significantly better PFS in patients with BM.^9^ In our recent study on the efficacy of three EGFR‐TKIs in patients with *EGFR*‐mutation‐positive advanced NSCLC,^14^ we found that patients who received afatinib had longer PFS and marginally longer OS than patients who received gefitinib. However, that study did not show a survival benefit in a subgroup of patients with BM, so we further analyzed whether afatinib and reversible EGFR‐TKIs would yield different survival durations in a subgroup of patients without BM in this study, enrolling more patients for a pooled analysis. We found that afatinib provided significantly longer PFS and OS durations than those indicated by the pooled data for erlotinib and gefitinib. A similar result was also found in other clinical trial, the phase III ARCHER 1050 trial, which excluded patients with BM. Mok *et al*. compared an irreversible EGFR‐TKI, dacomitinib, with a reversible EGFR‐TKI, gefitinib, in treatment‐naïve patients with *EGFR*‐mutant NSCLC, finding better PFS with dacomitinib than with gefitinib (14.7 vs. 9.2 months; HR, 0.59; 95% CI: 0.47 to 0.74; *P* < 0.001).^11^ The final OS analysis in that study also indicated a significant improvement with dacomitinib versus gefitinib (median OS, 34.1 vs. 26.8 months; HR, 0.76; 95% CI: 0.582 to 0.993; *P* = 0.0438).^12^ Furthermore, in a subgroup analysis of the LUX‐Lung 7 study, patients without BM were found to have a lower HR (0.81, 95% CI:0.61–1.07) than patients with BM (1.16, 95% CI: 0.61–2.21), a result which implied the trend of an OS benefit of afatinib in patients without BM. Therefore, we thought that the second‐generation EGFR‐TKIs afatinib and dacomitinib could potentially provide better PFS and OS in patients without BM that is not inferior to those of first‐line osimertinib.^8^, ^22^

The impact of common *EGFR* mutations, including exon 19 deletions and L858R substitutions, on responses to EGFR‐TKIs has remained controversial. In a randomized controlled trial of erlotinib versus gefitinib in advanced NSCLC patients harboring *EGFR* exon 19 or 21 mutations, patients were enrolled regardless of their line of treatment in order to determine whether erlotinib is superior to gefitinib in terms of response and survival. The patients with *EGFR* exon 19 mutations had a superior median OS compared to those with exon 21 mutations (22.9 vs. 17.8 months, HR 0.71, *P* = 0.022),^23^ a result which was similar to that of another meta‐analysis.^24^ In the current study, there was no significant difference in PFS or OS between the afatinib group and the gefitinib group. Using Cox proportional hazards regression to adjust for possible confounders, we also found that the mutation type was not a significant determinant of PFS or OS (Table 2). However, in the subgroup analysis of patients with L858R substitutions, the patients in the afatinib group had significantly better PFS and OS than the patients in the erlotinib or gefitinib group. The basic demographic and clinical characteristics of the patients with L858R mutations without brain metastases were also reviewed. Using Fisher's exact test, we found that the patients who received the second‐generation EGFR‐TKI as first‐line therapy had better performance statuses, although the difference was not statistically significant (Table S5). This finding was similar to the LUX‐Lung 7 study finding of a higher difference in the response rates to afatinib and gefitinib in patients harboring L858R substitutions.^10^ Another similar result was the ARCHER 1050 study finding that the OS benefit of dacomitinib was more significant in patients harboring L858R substitutions.^25^ Taken together, these findings indicate that the irreversible EGFR‐TKIs might provide greater survival benefits among patients harboring L858R substitutions.

In this study, there was no significant difference in the percentage of patients who developed T790M mutations (25%) in the irreversible EGFR‐TKI group compared to the reversible EGFR‐TKIs group. In addition, the T790M mutation rate was lower than those in previous clinical trials but similar to those in previous real‐world studies since the patients did not undergo rebiopsy for various reasons.^4^, ^26^ Furthermore, most of the patients received subsequent therapies, and the percentages of patients who received subsequent first‐line, second‐line, and third‐line therapies were also similar between the two groups of patients (Table S4).

**Figure 5:**
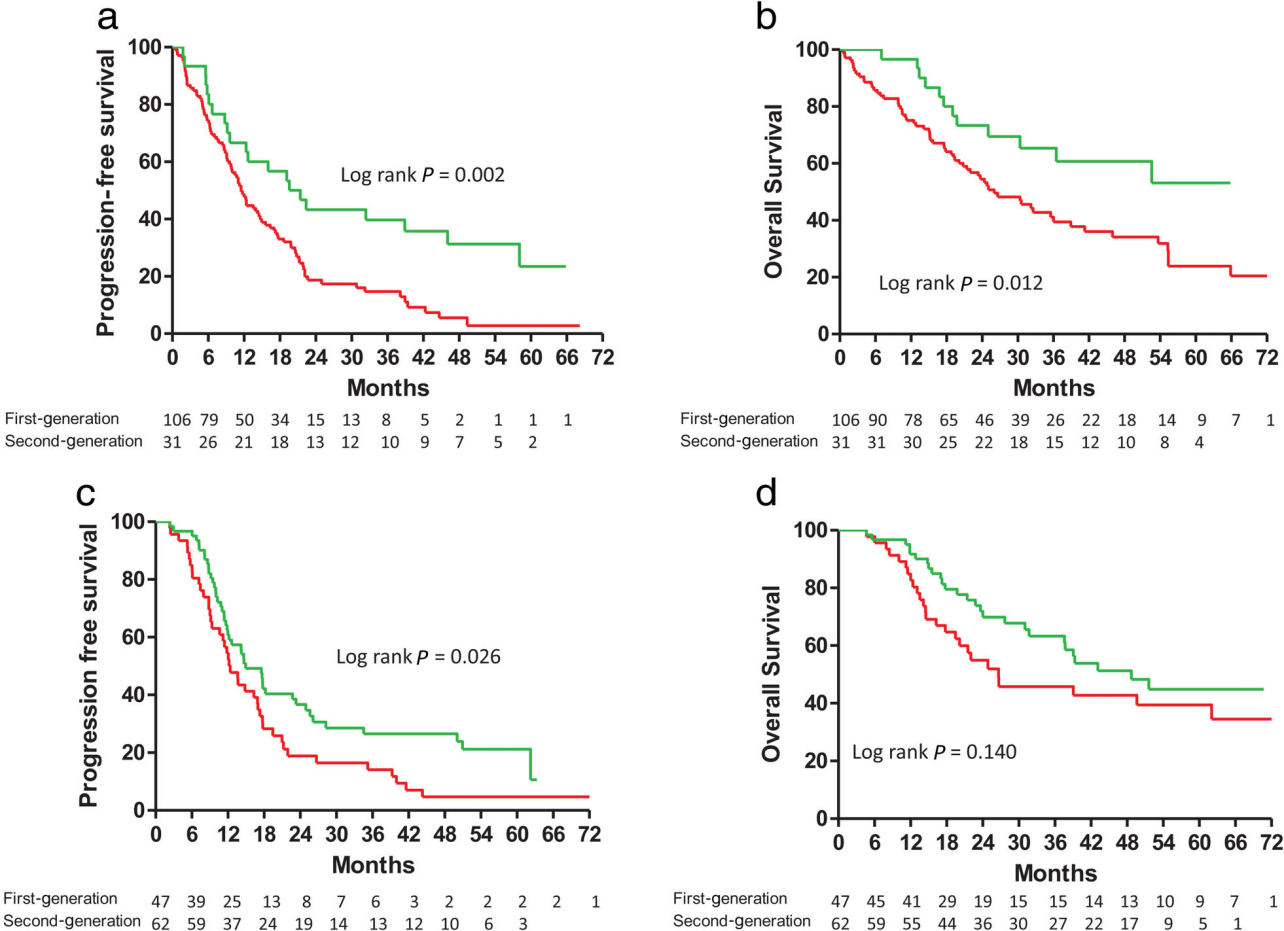
(**a**) Progression‐free survival (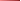) First‐generation 11.5 (5.8–21.3) (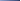) Second‐generation 21.0 (8.8–58.1); (**b**) Overall survival in patients with advanced stage non‐small cell lung cancer and L858R mutations, but without brain metastases, treated with first‐ or second‐generation EGFR‐TKIs (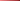) First‐generation 26.6 (13.1–NR) (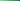) Second‐generation 52.6 (22.8–NR); (**c**) Progression‐free survival (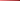) First‐generation 12.3 (7.9–21.0) (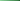) Second‐generation 14.9 (10.0–50.1); and (**d**) Overall survival in patients with advanced stage non‐small cell lung cancer and exon 19 deletions, but without brain metastases, treated with first‐ or second‐generation EGFR‐TKIs (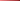) First‐generation 26.6 (14.1–NR) (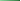) Second‐generation 48.7 (22.7–NR).(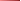)(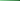)

This study had several limitations. First, because it was a single‐center retrospective study, there were significant differences in the characteristics of the two groups. The patients in the first‐generation EGFR‐TKI group had poorer performance statuses than those in the second‐generation EGFR‐TKI group, which might have interfered with the study results. However, using the Cox proportional hazard model to adjust for potential confounding factors, we found that treatment with afatinib was still an independent predictor for better PFS and OS. Second, a higher proportion of the patients receiving afatinib harbored exon 19 deletions. Another real‐world study, however, also showed similar differences,^4^ and one possible explanation is that the favorable OS of patients with exon 19 deletions found in a previous pooled analysis may be affecting physicians' decisions about using afatinib as a first‐line treatment.^7^ Furthermore, the PFS durations for patients harboring exon 19 deletions and L858R mutations were similar in a previous study.^26^ In the current study, however, the subgroup analysis of the patients with exon 19 deletions showed that afatinib provided better PFS but not OS (log‐rank test, *P* = 0.026 and 0.140, respectively, Fig 5c,d). However, using Cox proportional hazards regression to adjust for possible confounders, it was found that there was no significant difference in PFS or OS between the irreversible EGFR‐TKI group and the reversible EGFR‐TKI group overall (Table S2). However, differences in PFS and OS between the irreversible and reversible EGFR‐TKIs were found in patients with L858R substitutions. The higher proportion of exon 19 deletions in the irreversible EGFR‐TKI group thus did not affect the main conclusions of this study. Third, while all the patients underwent brain imaging at the time of the initial diagnosis or at recurrence, the brain imaging was conducted based on the occurrence of symptoms rather than according to a specific schedule, so we might have missed asymptomatic BM, which would have caused us to overestimate the PFS and OS. However, as the same follow‐up schedule was applied to each group of patients, differential bias would not have been generated.

In conclusion, the irreversible EGFR‐TKI afatinib provided better PFS and OS than the reversible EGFR‐TKIs gefitinib and erlotinib, and its efficacy may not have been inferior to that of osimertinib in the FLAURA study.^8^, ^9^ Moreover, in patients without BM, afatinib may provide greater benefits given its better hazard ratio. However, further large‐scale prospective studies are needed to compare the effects of afatinib and osimertinib in patients without BM.

## Disclosure

The authors declare no potential conflicts of interest with respect to the research, authorship, and/or publication of this article.

## Supporting information


**Table S1** Demographic and clinical characteristics of patients without brain metastases, subdivided by those treated with the first‐generation EGFR‐TKIs and those treated with the second‐generation EGFR‐TKI.
**Table S2.** Cox model subgroup analysis results indicating progression‐free survival and overall survival of patients with exon 21 L858R mutations.
**Table S3.** Cox model subgroup analysis results indicating progression‐free survival and overall survival of patients with exon 19 deletions.
**Table S4.** Subsequent therapies of patients without brain metastases, subdivided by those treated with the first‐generation EGFR‐TKIs and those treated with the second‐generation EGFR‐TKI.
**Table S5**. Demographic and clinical characteristics of patients with L858R mutations, but without brain metastases, subdivided by those treated with the first‐generation EGFR‐TKIs and those treated with the second‐generation EGFR‐TKI.
