# Supplementary material for: First‐line treatment with irreversible tyrosine kinase inhibitors associated with longer OS in EGFR mutation‐positive non‐small cell lung cancer
Source: Thorac Cancer. 2020 Dec 18;12(3):287–96. doi: 10.1111/1759-7714.13462 (PMC7862787; doi:10.1111/1759-7714.13462)
Supplement: Supplementary file 1 — Table S1 Demographic and clinical characteristics of patients without brain metastases, subdivided by those treated with the first‐generation EGFR‐TKIs and those treated with the second‐generation EGFR‐TKI. Table S2. Cox model subgroup analysis results indicating progression‐free survival and overall survival of patients with exon 21 L858R mutations. Table S3. Cox model subgroup analysis results indicating progression‐free survival and overall survival of patients with exon 19 deletions. Table S4. Subsequent therapies of patients without brain metastases, subdivided by those treated with the first‐generation EGFR‐TKIs and those treated with the second‐generation EGFR‐TKI. Table S5. Demographic and clinical characteristics of patients with L858R mutations, but without brain metastases, subdivided by those treated with the first‐generation EGFR‐TKIs and those treated with the second‐generation EGFR‐TKI. [file TCA-12-287-s001.docx]

**Supplementary Table 1.** Demographic and clinical characteristics of patients without brain metastases, subdivided by those treated with the first-generation EGFR-TKIs and those treated with the second-generation EGFR-TKI.

|  | **First-generation**  **(*N* = 153)** | **Second-generation**  **(N = 93)** | ***P* value** |
| --- | --- | --- | --- |
| **Age** | 69.8 [60.6-76.5] | 69.0 [60.9-78.7] | 0.133 |
| **Sex, *n* (%)**  **Male**  **Female** | 60 (39.2%)  93 (60.8%) | 40 (43.0%)  53 (57.0%) | 0.557 |
| **Tumor size, *n* (%)**  **≥3cm**  **<3cm**  **NA** | 97 (63.4%)  38 (24.8%)  18 (11.8%) | 62 (66.7%)  25 (26.9%)  6 (6.4%) | 0.394 |
| **Nodal involvement, *n* (%)**  **N0**  **N1/N2/N3** | 27 (17.6%)  126 (82.4%) | 18 (19.4%)  75 (80.6%) | 0.737 |
| **Stage, n (%)**  **Recurrence**  **Newly-diagnosed** | 24 (15.7%)  129 (84.3%) | 16 (17.2%)  77 (82.8%) | 0.754 |
| **ECOG PS, *n* (%)**  **0-1**  **≥2** | 133 (86.9%)  20 (13.1%) | 91 (97.8%)  2 (2.2%) | 0.004 |
| **EGFR mutation, n (%)**  **Del 19**  **L858R** | 47 (30.7%)  106 (69.3%) | 62 (66.7%)  31 (33.3%) | <0.001 |

ECOG, Eastern Cooperative Oncology Group; EGFR, epidermal growth factor receptor; PS, performance status; NA, not applicable.

**Supplementary Table 2.** Cox model subgroup analysis results indicating progression-free survival and overall survival of patients with exon 21 L858R mutations.

|  |  | **Progression-free survival** | | **Overall survival** | |
| --- | --- | --- | --- | --- | --- |
|  |  | HR (95% CI) | *p* value | HR (95% CI) | *p* value |
| Age | ≥60 versus <60 | 0.99 (0.61~1.59) | 0.952 | 1.37 (0.76~2.46) | 0.291 |
| Sex | Male versus female | 1.55 (1.06~2.26) | 0.025 | 1.67 (1.04~2.68) | 0.033 |
| ECOG PS | ≥2 versus <2 | 2.55 (1.41~4.60) | 0.002 | 4.39 (2.20~8.74) | <0.001 |
| Tumor size | >3cm versus <3cm | 1.73 (1.03~2.90) | 0.039 | 2.63 (1.31~5.26) | 0.006 |
| Nodal involvement | Positive versus negative | 1.77 (1.02~3.07) | 0.044 | 1.54 (0.80~2.97) | 0.195 |
| Recurrence | Newly-diagnosed versus recurrence | 0.77 (0.43~1.39) | 0.392 | 0.96 (0.45~2.02) | 0.906 |
| Treatment | Second-generation versus first-generation | 0.53 (0.32~0.96) | 0.011 | 0.49 (0.26~0.93) | 0.028 |

ECOG, Eastern Cooperative Oncology Group; EGFR, epidermal growth factor receptor; PS, performance status.

**Supplementary Table 3.** Cox model subgroup analysis results indicating progression-free survival and overall survival of patients with exon 19 deletions.

|  |  | **Progression-free survival** | | **Overall survival** | |
| --- | --- | --- | --- | --- | --- |
|  |  | HR (95% CI) | *p* value | HR (95% CI) | *p* value |
| Age | ≥60 versus <60 | 0.85 (0.52~1.38) | 0.508 | 1.31 (0.65~2.66) | 0.456 |
| Sex | Male versus female | 1.37 (0.89~2.11) | 0.160 | 1.61 (0.92~2.84) | 0.098 |
| ECOG PS | ≥2 versus <2 | 2.42 (0.90~6.49) | 0.079 | 8.32 (2.18~31.85) | 0.002 |
| Tumor size | >3cm versus <3cm | 1.04 (0.62~1.72) | 0.891 | 1.21 (0.60~2.43) | 0.592 |
| Nodal involvement | Positive versus negative | 2.32 (1.22~4.41) | 0.011 | 3.20 (1.25~8.18) | 0.015 |
| Recurrence | Newly-diagnosed versus recurrence | 1.27 (0.66~2.44) | 0.475 | 1.01 (0.42~2.46) | 0.984 |
| Treatment | Second-generation versus first-generation | 0.68 (0.44~1.05) | 0.081 | 0.47 (0.16~1.37) | 0.164 |

ECOG, Eastern Cooperative Oncology Group; EGFR, epidermal growth factor receptor; PS, performance status.

**Supplementary Table 4.** Subsequent therapies of patients without brain metastases, subdivided by those treated with the first-generation EGFR-TKIs and those treated with the second-generation EGFR-TKI.

|  | First-generation (N=153) | Second-generation (N=93) | P value |
| --- | --- | --- | --- |
| Presence of T790M  Osimertinib in subsequent therapies | 35 (22.9%)  29 (19.0%) | 17 (18.3%)  14 (15.1%) | 0.392  0.435 |
| First subsequent therapy  Cytotoxic chemotherapy  Other EGFR-TKI  Osimertinib  Immunotherapy  Clinical trial | 82 (53.6%)  57  3  17  0  5 | 47 (50.5%)  34  1  10  0  2 | 0.886 |
| Second subsequent therapy  Cytotoxic chemotherapy  Other EGFR-TKI  Osimertinib  Immunotherapy  Clinical trial | 48 (31.4%)  18  19  6  2  3 | 27 (29.0%)  15  9  1  1  1 | 0.947 |
| Third subsequent therapy  Cytotoxic chemotherapy  Other EGFR-TKI  Osimertinib  Immunotherapy  Clinical trial | 25 (16.3%)  10  7  7  1  0 | 12 (12.9%)  4  6  2  0  0 | 0.360 |

EGFR-TKI, epidermal growth factor receptor-tyrosine kinase inhibitor.

Supplementary Table 5. Demographic and clinical characteristics of patients with L858R mutations, but without brain metastases, subdivided by those treated with the first-generation EGFR-TKIs and those treated with the second-generation EGFR-TKI.

|  | **First-generation**  **(*N* = 106)** | **Second-generation**  **(N = 31)** | ***P* value** |
| --- | --- | --- | --- |
| **Age** | 69.1 [63.0-78.1] | 67.3 [61.3-77.2] | 0.737 |
| **Sex, *n* (%)**  **Male**  **Female** | 43 (40.6%)  63 (59.4%) | 12 (38.7%)  19 (61.3%) | 0.853 |
| **Tumor size, *n* (%)**  **≥3cm**  **<3cm**  **NA** | 69 (65.1%)  24 (22.6%)  13 (12.3%) | 22 (71.0%)  8 (25.8%)  1 (3.2%) | 0.342 |
| **Nodal involvement, *n* (%)**  **N0**  **N1/N2/N3** | 18 (17.0%)  88 (83.0%) | 6 (19.3%)  25 (80.7%) | 0.760 |
| **Stage, n (%)**  **Recurrence**  **Newly-diagnosed** | 19 (17.9%)  87 (82.1%) | 2 (6.5%)  29 (93.5%) | 0.119 |
| **ECOG PS, *n* (%)**  **0-1**  **≥2** | 87 (82.1%)  19 (17.9%) | 30 (96.8%)  1 (3.2%) | 0.078 |

ECOG, Eastern Cooperative Oncology Group; PS, performance status; NA, not applicable.
